# Delirium in trauma patients: a 1-year prospective cohort study of 2026 patients

**DOI:** 10.1007/s00068-021-01603-5

**Published:** 2021-02-04

**Authors:** Justus Marquetand, Samuel Gehrke, Leonie Bode, Simon Fuchs, Florian Hildenbrand, Jutta Ernst, Roland von Känel, Soenke Boettger

**Affiliations:** 1 Department of Consultation-Liaison Psychiatry and Psychosomatic Medicine, University Hospital Zurich, University of Zurich, Zurich, Switzerland; 2 Institute of Nursing Science, University Hospital Zurich, University of Zurich, Zurich, Switzerland; 3 Department of Epileptology, Hertie-Institute for Clinical Brain Research, University of Tubingen, Hoppe-Seyler-Str.3, 72076 Tubingen, Germany; 4 University Hospital Zurich, University Zurich, Zurich, Switzerland; 5 Department of Gastroenterology, University Hospital Zurich, University of Zurich, Zurich, Switzerland

**Keywords:** Trauma, Delirium, Disorders, Factors, Prospective

## Abstract

**Background:**

Delirium in trauma surgery is common, especially post-operatively, but medical characteristics, risk factors and residence post-discharge have not comprehensively been investigated in all trauma patients.

**Methods:**

Over 1 year, 2026 trauma patients were prospectively screened for delirium with the following tools: Delirium Observation screening scale (DOS), Intensive Care Delirium Screening Checklist (ICDSC) and a DSM (Diagnostic and Statistical Manual)-5, nursing tool (ePA-AC) construct. Risk factors—predisposing und precipitating—for delirium were assessed via multiple regression analysis.

**Results:**

Of 2026 trauma patients, 440 (21.7%) developed delirium, which was associated with an increased risk of assisted living (OR 6.42, CI 3.92–10.49), transfer to nursing home (OR 4.66, CI 3.29–6.6), rehabilitation (OR 3.96, CI 3.1–5.1), or death (OR 70.72, CI 22–227.64). Intensive care management (OR 18.62, CI 14.04–24.68), requirement of ventilation (OR 32.21, CI 21.27–48.78), or its duration (OR 67.22, CI 33.8–133.71) all increased the risk for developing delirium. Relevant predisposing risk factors were dementia (OR 50.92, CI 15.12–171.45), cardiac insufficiency (OR 11.76, CI 3.6–38.36), and polypharmacy (OR 5.9, CI 4.01–8.68).Relevant precipitating risk factors were brain edema (OR 40.53, CI 4.81–341.31), pneumonia (OR 39.66, CI 8.89–176.93) and cerebral inflammation (OR 21.74, CI 2.34–202.07).

**Conclusion:**

Delirium in trauma patients is associated with poor outcome as well as with intensive care management and various predisposing and/or precipitating factors. Three quarters of patients who had undergone delirium were not able to live independently at home any more.

**Supplementary Information:**

The online version contains supplementary material available at 10.1007/s00068-021-01603-5.

## Introduction

Delirium is an acute neuropsychiatric disorder, manifesting in fluctuating disorders of consciousness, attention and formal thinking [1]. Patients often also show additional symptoms, such as illusions, hallucinations, delusions, as well as agitation and disorders of emotionality [2].

Studies on delirium in surgical services have often focused on postoperative delirium, which is influenced by the type and duration of surgery [3, 4], requirement and duration of any postoperative intensive care management [5, 6], age [7] and any pre-existent illness [8]. In general, the more of the factors that are present or the longer they prevail, the higher the risk of postoperative delirium [9]. In other words, the older the patient is, the longer operations or ventilation take, and the more pre-existing illnesses, the higher the probability of postoperative delirium. These factors can be categorized into predisposing and precipitating ones [9–11]: predisposing factors exist prior to the development of delirium and include age, dementia or pre-existent illnesses. Precipitating factors represent newly emerging conditions causing delirium such as infections, fever or surgeries. In general, the more predisposing factors exist, the fewer precipitating factors are necessary for the development of delirium [12]. Previous studies have repeatedly emphasized the importance of identifying these factors as early as possible, because then treatment of delirium is more appropriate [9, 12, 13]. This particularly applies to intensive care units (ICUs), since the prevalence of delirium in trauma ICUs ranges between 19% [14] and 73% [15].

Although there are a number of studies on delirium in trauma surgery, important questions remain to be answered due to a lack of prospectively collected data, small patient samples, and pooled data. Specifically, the prevalence of delirium across different trauma surgery services is unknown regardless whether patients have been managed surgically. Moreover, it is unknown how and to what extent delirious trauma patients are different from other patients with delirium described in the literature, including predisposing and precipitating factors. Finally, the outcome of delirious trauma patients is underexplored.

To fill these gaps in knowledge, we performed a 1-year prospective study including all trauma patients in a tertiary university hospital. The objective of the study was 1- to assess the sociodemographic characteristics of delirious trauma patients, 2- to identify predisposing and precipitating risk factors for delirium, and 3- to compare the results with the "general" delirium described in the literature. Novel insight in these aspects could inform future studies to improve the management or advanced care planning of delirious trauma patients.

## Methods

### Study design, patients and procedures

**Fig. 1:**
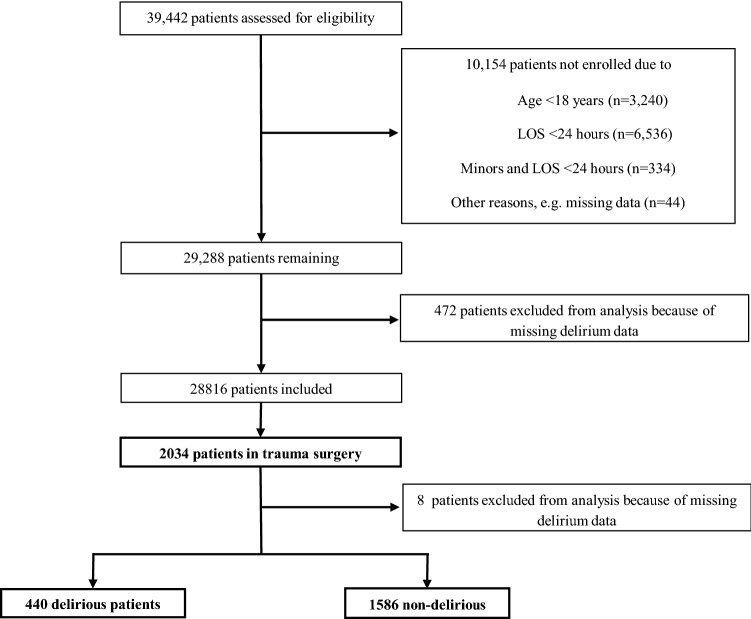
Screening algorithm of the Delir-Path

As part of a local delirium detection initiative (DelirPath, **D**etect **E**valuate Contro**l I**npatient **R**isk factors, **P**revent **A**nd **T**reat **H**ospital Acquired Deliriums) at the University Hospital Zurich, a tertiary care center, 39,442 patients were prospectively screened for delirium within 1 year (January 1st to December 31st 2014). Patients were excluded if 18 years or younger, length of stay (LOS) less than 1 day and data were missing, including the electronic patient’s assessment, leaving 28,816 eligible patients. Of these, 2034 were trauma patients (admitted through a trauma service), of whom 8 had to be excluded from further analysis due to missing or incomplete data, leaving 2026 patients for the present analysis (Fig. 1). Of these, 440 patients developed a delirium. The decision whether a patient was admitted to traumatology was made by the respective traumatologist. Every patient who was not admitted as an emergency but with an appointment was considered to be elective. The diagnoses were based on the encoded ICD-10 diagnoses. During the stay in the intensive care unit, the simplified acute severity score II (SAPS-II) was assessed. The SAPS-II is a well-established score for assessing the severity of disease in intensive care patients. It is composed of 12 different physiological parameters and has a maximum total score of 174 points. The higher the score [16], the higher the probability of mortality, e.g., a score of 20 points is associated with a mortality risk of about 4%, a score of 40 points with about 25–30%.

### Measurements and determination of delirium

For the determination of a delirium, a set of scales was used: 1- The Delirium Observation Screening Scale (DOS, cut-off ≥ 3) [17], 2- the Intensive Care Delirium Screening Checklist (ICDSC, cut-off ≥ 4) [18], and 3- a DSM-5-criteria—disturbances in consciousness, attention and cognition-based construct derived a nursing instrument, the Ergebnisorientiertes PflegeAssessment Acute-Care (ePA-AC) [19, 20]. This delirium screening approach was briefly validated [20, 21]; this construct detected 91% of delirium diagnoses as determined by the gold-standard of, an assessment by the consultation-liaison psychiatry service, correctly. Furthermore, this construct was tested against the validated DOS and ICDSC approach and achieved perfect agreement (Cohen’s κ 0.83, *p* < 0.001). On regular floors, patients  ≥ 80 years were screened daily with DOS and ePA-AC. On intensive care units (ICU), ICDSC was conducted three times per day; sedation holidays were not part during the ICU-treatment. Patients below 80 years were not routinely screened for delirium at hospital admission, but the delirium scales were applied in cases of clinical suspicion and a consultation psychiatry service was usually involved. DOS, ICDSC and ePA-AC were conducted by nursing staff and continued until remission of delirium was apparent. The criteria for remission were normalization of delirium scales and the clinical evaluation. In the case of a follow-up surgery or a return to intensive care, the delirium scales were restarted. The nursing staff had been trained in a four-hour course with achievement tests. Additionally, part of the education was performed with discussion of case reports, state-of-the-art lessons on epidemiology and knowledge about delirium, including diagnostic criteria.

The DOS is a 13-item scale validated to indicate delirium according to DSM-IV criteria [17]. Items include disturbances of consciousness (1), attention (2–4), thought processes (5 and 6), orientation (7 and 8), memory (9), psychomotor behavior (10, 11 and 13), and affect (12). Symptoms are rated on a scale (0–1) as not existent (0), sometimes to always existent (1), and unable to assess (–). The cut-off score for delirium is  ≥ 3 and values were aggregated throughout recordings. This approach proved to be valid and correctly identified 91% of delirium diagnoses as determined by the consultation-liaison psychiatry service.

The ICDSC is a screening instrument with eight items based on the DSM-IV criteria specifically designed for the intensive care setting with two points [18]: Absent or present. This scale was designed for patients with limited communication abilities such as intubated patients. The items include the assessment of 1—consciousness (comatose, soporose, awake, or hypervigilant), 2—orientation, 3—hallucinations or delusions, 4—psychomotor activity, 5—inappropriate speech or mood, 6—attentiveness, 7—sleep–wake cycle disturbances and 8—fluctuation of symptomatology. The maximum score is eight; scores of more than three indicate the presence of delirium. Each item is rated on the patient’s behavior over the previous eight.

The ePA-AC is a nursing instrument administered daily assessing mobility, personal care and dressing, feeding, elimination, cognition and alertness, communication and interaction, sleeping, breathing, pain, pressure ulcers and wounds [19]. Items are rated on scales from either 0—absent to 1—present, or from 1 to 4, most commonly representing 1—no ability, 2—substantial impairment, 3—mild impairment, and 4—full ability; or for consciousness 1—comatose, 2—soporose, 3—somnolent, and 4—awake and alert; or for orientation, 1—no quality, 2—single quality, 3—two qualities, and 4—fully oriented. For most items, the inability to assess is coded as 9. A cut-off score of the ePA-AC for the delirium does not exist, but a qualitative evaluation of the ePA-AC was considered in the assessment of whether or not a delirium is present; for example, delirious patients are usually not able to groom their own hair [20].

DOS, ICDSC and ePA-AC values, as well as medical data, were obtained from the electronic medical chart (Klinikinformationssystem, KISIM, CisTec AG, Zurich). Predisposing and precipitating factors were assessed on the basis of the ICD-10 coded diagnoses. This study was approved by the ethics committee of the Canton of Zurich (KEK-ZH-Nr. 2012-0263). A waiver of informed consent was obtained from the committee. Our reporting is in line with the STROBE (strengthening the reporting of observational studies in epidemiology)-statement [22].

### Statistical analysis

Data were analyzed with the Statistical Package for the Social Sciences (SPSS) version 25 and R statistical software version 3.5.0 for Windows. Descriptive characteristics are summarized depending on parametric properties using means and standard deviations or medians and interquartile ranges for continuous variables, and percentages for categorical variables.

The data were tested with Shapiro–Wilk’s test for distribution of normality. Inter-group differences for continuous variables were computed using Student’s *t*-test and Mann–Whitney *U*-test depending on their parametric properties, and for categorical variables with Pearson’s-*χ*^2^ test.

In a first step, the delirium construct based on DSM-5 was tested and its agreement with the validated approach—a DOS cut-off ≥ 3 or ICDSC ≥ 4—were determined with Cohen’s κ as measure of concordance. A value > 0.80, indicated perfect agreement [23].

Then, simple logistic regressions were calculated to determine the prevalence rates of delirium for medical characteristics, and their respective odds ratios (OR) and corresponding confidence intervals (CIs). Multiple regression models were computed with their respective ORs and CIs, based on the results of the simple logistic regressions models, by entering variables with a *p*-value < 0.15. The model was verified with Cox–Snell’s and Nagelkerke’s *r*^2^.

For all inferential tests, two-tailed tests were chosen and the significance level alpha (*α*) was set at *p* < 0.05.

## Results

### Sociodemographic characteristics of delirious patients

**Fig. 2:**
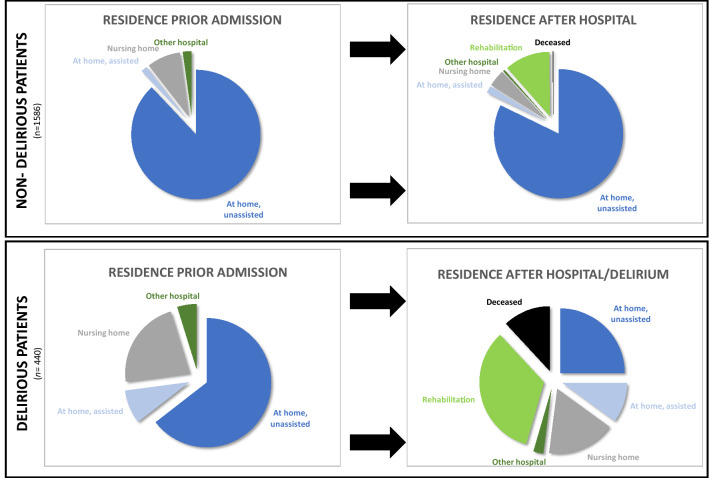
Visualization of relevant sociodemographic aspects of non-delirious (left) and delirious patients (right)

A total of 440 patients developed a delirium corresponding to a prevalence of 21.7%. The characteristics of delirious and non-delirious patients are shown in Table 1 and Fig. 2. Prior to admission, delirious patients were more commonly dependent on assistance at home (OR 6.0) or resided in nursing homes (OR 2.98) representing a greater risk of admission for the frail. Emergency admissions (OR 1.98) and requirement for intensive care management (OR 18.62) were more common, and even increased once ventilated (OR 32.21), in particular for > 24 h (67.22), albeit with considerably wide confidence intervals. Delirious patients were operated significantly more often. The LOS was longer in delirious than in non-delirious patients. Post discharge, approximately three quarters of patients were unable to reside independently at home (OR 0.07), rather than being dependent on assistance at home (6.42), transferred to a nursing home (4.66), or to rehabilitation (OR 3.96) or deceased (OR 70.72). Although the age was different, the factor old age was included as a covariate in the multiple regression. According to the literature, old age refers to an age > 65 years [9]. The results of the multiple regression regarding predisposing and precipitating factors is consequently corrected for old age.

**Table 1:** Sociodemographic and medical characteristics of delirious patients

	Non-delirious patients (*n* = 1586)	Delirious patients (*n* = 440)	P, OR, CI
Age in years	51.7, 20 / 51, 34	63.6, 20.8 / 67, 34	< 0.001, –, –
Gender in %			
Male	57.3	60.9	0.169, 1.16, 0.94–1.44
Female	42.7	39.1	0.169, 0.86, 0.69–1.07
Residence prior admission in %			
At home, unassisted	87.8	64.5	< 0.001, 0.27, 0.21–0.34
At home, assisted	1.5	8.4	< 0.001, 6.0, 3.53–10.1
Nursing home	8.3	22.3	< 0.001, 2.98, 2.25–4
Other hospital	2.4	4.8	0.009, 2.04, 1.19–3.52
Admission in %			
Emergency	65.7	79.1	< 0.001, 1.98, 1.54–2.54
Elective	34.3	20.9	< 0.001, 0.2, 0.14–0.3
Intensive care treatment			
In %	5.8	53.4	< 0.001, 18.62, 14.04–24.68
Treatment duration			
In hours	53.2, 84.6/28, 39	167.7, 202.9 / 84, 150	< 0.001, –, –
Ventilation during ICU			
Ventilated	1.8	37.5	< 0.001, 32.21, 21.27–48.78
Ventilated > 24 h	0.6	27.7	< 0.001, 67.22, 33.8–133.71
Ventilated in hours	10.3, 39.3/10, 8	88.1, 154.2 / 24, 96	< 0.001, –, –
Number of			
Diagnoses	5.7, 5.1/4, 5	6.3, 4.7/5, 6	< 0.001, –, –
Surgeries	12.5, 9.5/9, 10	16.9, 12.7/12, 15	< 0.001, –, –
SAPS-II	26.6, 15.7 / 23.6, 18	40.9, 22.7 / 37, 26	< 0.001, –, –
Length of stay (LOS)			
Days	11.8, 10.4/10, 8	16.1, 13.9/12, 13	< 0.001, –, –
Residence after hospital/delirium in %			
At home, unassisted	82.2	25	< 0.001, 0.07, 0.06–0.09
At home, assisted	1.7	10	< 0.001, 6.42, 3.92–10.49
Nursing home	4.2	17	< 0.001, 4.66, 3.29–6.6
Other hospital	0.4	2.5	< 0.001, 6.75, 2.48–18.36
Rehabilitation	11.3	33.6	< 0.001, 3.96, 3.1–5.1
Deceased	0.2	11.9	< 0.001, 70.72, 22–227.64

*Mean, standard deviation (SD)/median, interquartile range (IQR); simplified acute severity score-II (SAPS-II), which has a maximum total score of 174 points

### Predisposing factors for delirium

Overall, predisposing factors for the development of delirium were related to diseases of the nervous, cardiac and endocrinological system (see Table 2); also, polypharmacy was associated with a high risk for delirium (OR 5.9). The most relevant predisposing factor was dementia, causing a 50.92-fold increased risk of developing delirium. Confidence intervals were wide for dementia, cardiac insufficiency and cardiomyopathy.

**Table 2:** Predisposing and precipitating risk factors

*n* = 2026	B (SE)	Exp(B)	CI	Sig
Predisposing factors Dementia Cardiac insufficiency Polypharmacy Cardiomyopathy Valvular heart disease Diabetes mellitus type II Epilepsy Precipitating factors Brain edema Pneumonia Cerebral inflammation Sepsis/SIRS Intracranial hemorrhage Cardiac arrest Stroke Thrombosis Myocardial infarction Constant	3.93 (0.62) 2.47 (0.6) 1.77 (0.2) 1.47 (0.99) 1.37 (0.4) 1.08 (0.22) 1.02 (0.33) 3.7 (1.09) 3.68 (0.76) 3.08 (1.14) 2.86 (0.53) 2.55 (0.7) 2.49 (0.6) 2.06 (0.57) 1.18 (0.56) 0.54 (0.23) − 2.03 (0.78)	50.92 11.76 5.9 4.33 3.94 2.95 2.79 40.53 39.66 21.74 17.51 12.75 12.1 7.82 3.24 1.71 0.13	15.12–171.45 3.6–38.36 4.01–8.68 0.61–30.653 1.79–8.7 1.94–4.5 1.45–5.34 4.81–341.31 8.89–176.93 2.34–202.07 6.21–49.4 3.23–50.38 1.09–134.05 0.64–96.26 1.09–9.67 1.1–2.68 –	< 0.001 < 0.001 < 0.001 0.142 0.001 < 0.001 0.002 0.001 < 0.001 0.007 < 0.001 < 0.001 0.042 0.108 0.035 0.018 –

B represents the unstandardized beta (*B*) and (SE) the standard error for the unstandardized beta. Exp (B) is the exponentiation of the B coefficient, which is an odds ratio

### Precipitating factors for delirium

Acute inflammatory diseases and brain edema significantly increased the risk of delirium. The most relevant precipitating risk factors were edema of the brain (OR 40.53), followed by pneumonia (OR 39.66) and cerebral inflammation (OR 17.51).

## Discussion

The delirium is a frequently overlooked acute neuropsychiatric disorder, which is often the result of a potentially life-threatening condition [9, 21]. Delirious patients are more likely to develop complications [15], show an increased mortality rate [24], and the direct as well as indirect costs of a delirium are considerable [25], which makes studies on delirium necessary.

### Summary of main findings—medical characteristics and risk factors

The prevalence of delirium in trauma patients was 21.7%, which includes both intensive care and patients on regular units. Those patients developing delirium during their hospital stay were more likely to require assistance at home or lived in a nursing home prior to admission, which occurred more frequently as an emergency. Notably, the risk for developing delirium increased with the requirement for intensive care and assisted ventilation. Regarding assisted ventilation, the risk for delirium doubled, when a ventilation duration was more than 24 h. It is evident that the influence of the number of operations has an influence on the development of delirium, so the number of operations was significantly higher in the delirium patients. At discharge, delirium was associated with a high risk of mortality and approximately three quarters of all patients were dependent on assistance, i.e., assisted living or nursing home, or rehabilitation. In addition to known relevant predisposing factors such as dementia, further factors were identified: cardiac insufficiency or valvular heart disease, polypharmacy, diabetes mellitus type II, and epilepsy. With respect to precipitating factors, cerebral edema increased the risk for delirium. Furthermore, inflammatory diseases such as cerebral inflammation, sepsis-related disorders or pneumonia increased the risk for delirium.

### Comparison with the existing literature

Previous studies focused on delirium in surgical or trauma ICU patients [4, 6, 14, 15, 24] with no previous investigations describing trauma patients on both ICU and regular units. One prospective study [14] of 818 patients on the surgical ICU determined a prevalence rate of 11%, and – different to our results – age, gender, length of stay in hospital were not associated with delirium. Relevant precipitating factors were comparable and included pneumonia and infections (ORs 30.6 vs. 39.66 and 18.0, vs. 17.51) in that study. Previously reported delirium rates [14] were comparably lower as determined in our study and studies in the trauma ICU, and this might be an effect caused by different health systems. Previous studies reported a wide range of prevalence rates from 19% [4, 15] to 73% [15] and implied that delirium in critically ill patients is different from delirium on regular units [15].

### Implications

The causes, manifestations and outcomes of delirium vary with the underlying diseases; although this seems plausible, in this study on delirium in trauma patients, also admission mode, predisposing and precipitating factors for delirium and outcome in general were not favorable. The results of this study can contribute to potential strategies for future risk detection and management studies, as well as advanced care planning. Regarding advanced care planning, patients and their relatives can—in knowledge of the predisposing factors mentioned above (e.g., cardiac insufficiency)—nominate a substitute decision maker. In the case of a delirium, which often goes hand in hand with an inability to judge, the substitute decision maker can carry out the presumptive will of the patient, which has been proven to reduce the anxiety of the patient and their relatives*.* In addition, these findings once again confirmed that delirium is more common than expected and a potentially serious, life-threatening condition.

### Strengths and limitations

This study has several strengths; however, a few limitations have to be noted, too. The strengths are the -1 prospective nature of data collection and -2 overall large group sizes and -3 comprehensive description of medical, sociodemographic and clinical characteristics of delirious trauma patients. A novelty of this study is the investigation across all trauma patients and not only postoperative patients. The relationship between the severity of illness and development of delirium could not be assessed; although SAPS-II was assessed for the intensive care patients, no score for injury severity was obtained, so that no information on injury severity and delirium can be derived. In addition, it seems problematic that patients with traumatic brain injury (TBI) were categorized as delirious, because neither scores for TBI were collected nor current delirium scores are validated with respect to TBI. This circumstance could further bias the data. The collected data is from 2014 and may not be fully generalizable due to improved delirium prevention in recent years. The sample in this study was representative of a tertiary care center, so the generalizability to other health settings may be limited. Future studies are required to confirm these findings. Some confidence intervals were wide, which limits the interpretation of group differences.

## Conclusion

Delirium in trauma patients leads to loss of independence in three out of four patients and comes with high mortality. Several predisposing and precipitating risk factors should be recognized in time for earlier management of delirium.

## Supplementary Information

Below is the link to the electronic supplementary material.

Diagnostic clusters with their respective included diagnoses according to the International Statistical Classifications of Diseases and Related Health Problems 10th Revision (1CD-10) (DOCX 17 KB)

## Data Availability

The anonymised data and materials are stored locally and any raw data from the statistical analysis can be made available on reasonable request.

## Abbreviations

DSM-5: Diagnostic and statistical manual of mental disorders, 5th edition
DOS: Delirium observation screening scale
ePA-AC: Ergebnisorientiertes PflegeAssessment Acute-Care
ICDSC: Intensive care delirium screening checklist
ICU: Intensive care unit
IMC: Intermediate care unit
SAPS-II: Simplified acute severity score II
