# Supplementary material for: Delirium in trauma patients: a 1-year prospective cohort study of 2026 patients
Source: Eur J Trauma Emerg Surg. 2021 Feb 4;48(2):1017–24. doi: 10.1007/s00068-021-01603-5 (PMC9001539; doi:10.1007/s00068-021-01603-5)
Supplement: Supplementary file 1 — Diagnostic clusters with their respective included diagnoses according to the International Statistical Classifications of Diseases and Related Health Problems 10th Revision (1CD-10) (DOCX 17 KB) [file 68_2021_1603_MOESM1_ESM.docx]

***Supplement Table 1:*** Diagnostic clusters with their respective included diagnoses according to the International Statistical Classifications of Diseases and Related Health Problems 10^th^ Revision (1CD-10)

|  | **ICD-10-Chapter** |
| --- | --- |
| Dementias / degenerative cerebral disorders | F00 Alzheimer’s disease F01 Vascular dementias F02 Dementia due to elsewhere defined disorders F03 Neurodegenerative disorder  G30 Alzheimer’s disease G31-.0 Localized atrophies (frontal temporal dementia) G31-.1–2 Senile and alcohol-induced degenerations G31.8–9 Degenerations ned G32 Degenerations due to elsewhere defined disorders |
| Diseases of the neurological system | G00-09 Cerebral inflammation  G40 Epilepsies  G46 Stroke  G93.6 Brain edema |
| Intracerebral hemorrhage | I61–62 |
| Sepsis-related disorders | A40–41 Other sepsis, streptococcal B00.7 Herpetic sepsis R65 Systemic inflammatory response syndrome |
| Polypharmacy | Y57.9 |
| Diseseas of the cardiovascular system | I21 Myocardial infarction  I34-I37 Valvular heart disesase  I42 Cardiomyopathy  I46 Cardiac arrest  I50 Cardiac insufficiency  I80 Thrombosis |
| Diseases of the pulmonary system | J12-J18 Pneumonia |
| Diseases of the endocrinological system | E11 Diabetes mellitus type II |
